# Prospects for the use of artificial intelligence methods in autism spectrum disorders

**DOI:** 10.1192/j.eurpsy.2023.2195

**Published:** 2023-07-19

**Authors:** A. Sidenkova, V. Litvinenko

**Affiliations:** Psychiatry, Ural State Medical University, Yekaterinburg, Russian Federation

## Abstract

**Introduction:**

ASD is a heterogeneous group of pathological conditions.

**Objectives:**

Sensory Functions in Children with Autism

**Methods:**

A brief review of scientific publications is presented, formed by the search result for the keywords: autism spectrum disorders, artificial intelligence, machine learning.

**Results:**

Patients with ASD respond to sensory information hyper-reactively and hypo-reactively. Patients, regardless of age and severity of ASD, have atypical information processing patterns in all sensory modalities. Atypical processing of stimuli correlates with social, cognitive and communication disorders.

**Conclusions:**

To normalize sensory deviations, it is necessary to build corrective and prognostic models of the child’s connection with the environment. Machine learning models, Data Mining methods to make medical management decisions and develop personalized therapeutic strategies.

**Disclosure of Interest:**

None Declared

